# Supplementary material for: Association Between 24-Hour Movement Behaviors and Noncommunicable Chronic Diseases Among Adult and Older Adult Users of the Brazilian Community Health Promotion Program
Source: Healthcare (Basel). 2025 Aug 15;13(16):2016. doi: 10.3390/healthcare13162016 (PMC12385304; doi:10.3390/healthcare13162016)
Supplement: Supplementary file 1 [file healthcare-13-02016-s001.zip › healthcare-3754428-supplementary.pdf]

Supplemental Materials. Goodness-of-Fit Measures for Binary and Multinomial Logistic Regression Models.

Table S1. Model Fit Measures for Binary Logistic Regression with Dependent, Independent, and Covariate Variables.

Variables	AUC (IC95%)	Hosmer-Lemeshow	R^2 de Nagelkerke	VIF
**Hypertension**				
**PA**	0,77 (0,60 - 0,90)	0,74	0,42	1,2
**Sleep**	0,79 (0,64 - 0,92)	0,60	0,47	2,5
**Screen Time**	0,83 (0,69 - 0,91)	0,77	0,53	3,3
**Adherence to 24-hour movement behavior recommendations**	0,85 (0,65- 0,94)	0,53	0,58	2,3
**Diabetes**				
**PA**	0,75 (0,62 – 0,88)	0,87	0,43	2,8
**Sleep**	0,78 (0,66 – 0,90)	0,64	0,41	2,9
**Screen Time**	0,81 (0,70 – 0,89)	0,70	0,43	3,8
**Adherence to 24-hour movement behavior recommendations**	0,84 (0,68 – 0,93)	0,82	0,46	2,8
**Hypercholesterolemia**				
**PA**	0.74 (0.61 – 0.87)	0.67	0.51	2.4
**Sleep**	0.77 (0.65 – 0.89)	0.85	0.48	2.1
**Screen Time**	0.80 (0.69 – 0.88)	0.62	0.57	3.2
**Adherence to 24-hour movement behavior recommendations**	0.83 (0.67 – 0.92)	0.81	0.42	3.1
**Cardiovascular disease**				
**PA**	0.73 (0.60 – 0.86)	0.72	0.59	2.2
**Sleep**	0.76 (0.64 – 0.88)	0.69	0.51	1.5
**Screen Time**	0.79 (0.68 – 0.87)	0.55	0.60	2.8
**Adherence to 24-hour movement behavior recommendations**	0.82 (0.66 – 0.91)	0.83	0.49	1.9

Note. PA: physical activity; AUC – Area Under the ROC Curve; CI: confidence interval; VIF – Variance Inflation Factor

Table S2. Model Fit Measures for Multinomial Logistic Regression with Dependent, Independent, and Covariate Variables.

Two noncommunicable diseases		
**Variables**	**R^2 de Nagelkerke**	**VIF**
**PA**	0.45	1.8
**Sleep**	0.49	2.7
**Screen Time**	0.48	2.5
**Adherence to 24-hour movement behavior recommendations**	0.56	1.3
**≥ Three noncommunicable diseases**		
**PA**	0.43	3.2
**Sleep**	0.48	1.7
**Screen Time**	0.49	1.6.
**Adherence to 24-hour movement behavior recommendations**	0.47	2.8

Note. PA: physical activity; VIF – Variance Inflation Factor
